# Examining the efficacy of intravenous ibuprofen and meperidine for preventing post-operative shivering after laparoscopic cholecystectomy with general anesthesia

**DOI:** 10.25122/jml-2022-0040

**Published:** 2023-07

**Authors:** Ghodrat Akhavanakbari, Khatereh Isazadehfar, Masood Entezariasl, Aziz Kamran, Sahel Rezapour

**Affiliations:** 1 Department of Anesthesiology, Faculty of Medicine, Ardabil University of Medical Sciences, Ardabil, Iran; 2 Social Determinants of Health Research Center (SDHRC), Ardabil University of Medical Sciences, Ardabil, Iran; 3 Department of Social Medicine, Faculty of Medicine, Ardabil University of Medical Sciences, Ardabil, Iran; 4 Department of Health Education and Promotion, Faculty of Medicine, Ardabil University of Medical Sciences, Ardabil, Iran

**Keywords:** general anesthesia, shivering, intravenous ibuprofen, meperidine

## Abstract

Postoperative shivering is a common complication that can lead to increased postoperative complications. This study aimed to compare the effectiveness of intravenous ibuprofen and meperidine in preventing shivering following laparoscopic cholecystectomy. A total of 120 patients, aged 20-70 and classified as ASA I–II, were enrolled in this triple-blind clinical trial. The participants were randomly assigned to one of three groups: ibuprofen (800mg IV), meperidine (30 mg), or placebo (normal saline 2 ml), administered 30 minutes before the end of surgery. The occurrence of postoperative shivering was assessed and recorded at regular intervals (0, 5, 10, 15, 30, and 60 minutes after surgery). Additionally, postoperative pain levels were measured using a visual analog scale (VAS), sedation levels were evaluated using the Ramsay Sedation Scale (RSS), and the incidence of postoperative nausea and vomiting was documented. The prevalence and severity of postoperative shivering were not statistically significant between groups. The VAS was significantly lower in the meperidine group than the ibuprofen group throughout the study (p <0.001). The VAS was significantly lower in the ibuprofen group than the placebo group at 0 and 15 minutes after surgery. Although the incidence of nausea was slightly higher in the meperidine group, the difference was not statistically significant (p=0.75). Sedation scores were consistently lower in the ibuprofen group and higher in the meperidine group compared to the other groups (p<0.0001) The meperidine group had a significantly higher sedation score indicative of deep sleepiness (score of 4) than the other groups. Intravenous ibuprofen demonstrated comparable efficacy to meperidine in controlling shivering. Additionally, the incidence of nausea, vomiting, and sleepiness was lower in the intravenous ibuprofen group, suggesting it is a potential alternative to meperidine.

## INTRODUCTION

Postoperative shivering is a frequent and unpleasant complication experienced during the recovery phase from anesthesia, with reported incidences ranging from 5% to 60% in various studies [1-3]. The underlying causes of postoperative shivering are not clear, but evidence suggests that it may be attributed to the suppression of the body’s temperature control center in the hypothalamus by anesthetic drugs, body relaxation, operating room temperature, cold injectable serums, open body cavities that cause hypothermia, and spinal anesthesia. Sympathetic block-induced vasodilation also contributes to heat loss and vulnerability to shivering and hypothermia [4, 5].

Post-operative shivering can have significant physiological effects, including increased cardiac output, oxygen consumption, CO2 production, blood pressure, circulating catecholamines, intracranial and intraocular pressures, wound suture opening, wound infections, hospitalization time, ventilation of the lung, ocular pressure and surgical site elongation. These consequences result in increased patient pain, compromised monitoring accuracy (especially with pulse oximeters), and in rare cases, hypoxia and delayed awakening [2-4, 6, 7].

Pharmacological methods for treating shivering include using drugs such as meperidine, alfentanil, clonidine, physostigmine, ketanserin, and magnesium sulfate [8-10]. Each of these medications act through distinct mechanisms to mitigate postoperative shivering, yet they may also carry different side effects [11]. Meperidine is a widespread drug that directly affects the central nervous system (CNS) and can cause side effects, including nausea and vomiting, itching, hypotension, bronchospasm, bradycardia, pruritus, sedation, respiratory depression [6, 12-14]. Consequently, its administration necessitates careful monitoring, particularly to prevent respiratory depression and apnea [4], which pose a greater risk for elderly patients. Therefore, there is a need to explore alternative options for shivering prevention.

Ibuprofen, a non-steroidal anti-inflammatory drug with analgesic and antipyretic effects, is inexpensive, widely available, and associated with fewer complications [15-17]. We hypothesized that ibuprofen could effectively relieve postoperative pain and shivering without causing pruritus, respiratory problems, or hemodynamic instability, at least to a similar extent as meperidine. To test this hypothesis, we conducted a randomized, triple-blind, placebo-controlled study to compare the postoperative analgesic and anti-shivering effects of ibuprofen and meperidine in patients undergoing laparoscopic cholecystectomy under general anesthesia.

## MATERIAL AND METHODS

### Study design and participants

This triple-blind clinical trial study was registered at the Iranian Clinical Trial Registry (IRCT20171217037921N1) and approved by the Ethics Committee of Ardabil University of Medical Sciences (Ethics Code ARUMS. REC. 1396. 164). The study was conducted at Emam Khomeini Hospital in Ardabil, Iran, between 2017-2018, including 120 adult patients.

### Sample size calculation and participant selection

The sample size was estimated based on similar studies [18], considering an α value of 0.5, β value of 0.8, and a difference (d) of µ1-µ2=0.26. The final sample size was determined as 40 participants per group to account for potential dropouts. Participants aged 20 to 70 years, classified as American Society of Anesthesiologists (ASA) physical status I or II, and scheduled for laparoscopic cholecystectomy under general anesthesia were included. Patients with severe cardiovascular and respiratory instabilities, liver and kidney disorders, peptic ulcer, muscular disease, blood transfusion during surgery, or history of seizure, and surgeries exceeding 2 hours in duration were excluded.

### Interventions and anesthesia protocol

Participants were randomly assigned to one of the three groups: ibuprofen 800mg IV (Ibuprofen800, 800mg/8ml, Caspian, Rasht, Iran), meperidine 30 mg (Pethidine 100, 100mg/2ml, Caspian, Rasht, Iran) or normal saline as a placebo (8 ml). The allocation concealment was ensured using a random number generator, and the assignment results were placed into consecutively numbered sealed envelopes by a third person who was not involved in the trial. Neither the patients, the researcher responsible for data collection, nor the analyzer were aware of the assigned intervention, ensuring a triple-blind study design.

The three injections (ibuprofen, meperidine, and placebo) were prepared in identical syringes with a total volume of 2 ml. The investigator responsible for controlling and recording the clinical signs remained blinded to the specific drugs administered. The injections were administered 30 minutes prior to the conclusion of surgery.

Anesthesia was induced using fentanyl at 1 µg/kg, propofol at 2 mg/kg, and atracurium at 0.5 mg/kg, following a standardized protocol for all patients. Tracheal intubation was performed, and anesthesia was maintained by infusing propofol at 100 µg/kg/min. Muscular relaxation was maintained during the operation to facilitate intubation and optimize surgical conditions. Atracurium was administered as required, and patients were mechanically ventilated throughout the surgical procedure. All patients were monitored in the recovery room.

### Outcome measures and data collection

The primary outcome measure was the occurrence and severity of postoperative shivering, assessed using the grading scale developed by Crossley *et al*. [19] (Table 1). Secondary outcomes included postoperative nausea, vomiting, sedation evaluated using the Ramsay Sedation Scale (Table 2), and pain levels assessed using the Visual Analogue Scale (VAS). Incidences of nausea and vomiting were identified through direct patient assessment in the recovery room. Any occurrence of nausea, retching, or vomiting was recorded using the postoperative nausea and vomiting score (PONV score). No prophylactic measures for postoperative nausea and vomiting (PONV) were administered intraoperatively. All outcomes were recorded upon admission to the recovery room and 5, 10, 15, 30, and 60 minutes after entering.

**Table 1:** Patients level of shivering

Shivering grade	Specification of shivering	Symptoms
0	Without	-
I	Slight level	Insignificant but evident peripheral vasoconstriction
II	Medium level	The activity of muscles is seen only in one muscle group
III	Severe	The activity of muscles is seen in more than one muscle group, while it does not have generalized shivering
IV	Generalized level	Generalized shivering

**Table 2:** Scores of sedation in patients according to the Ramsay Sedation Scale

Score	Symptoms/ Description
1	- Anxious - Agitated - Restless
2	- Cooperative - Oriented to person - Tranquil
3	- The patient could respond to commands only
4	- Brisk reflexes to a light glabellar tap - Brisk reflexes to a loud auditory stimulus
5	- Sluggish response to a light glabellar tap - Sluggish response to loud auditory stimulus
6	- There is no response to light glabellar taps - There is no response to the loud auditory stimulus

### Statistical analysis

All data analysis was performed using SPSS software version 20. Descriptive statistics were presented as frequencies, percentages, and mean±standard deviation (SD). Categorical data were analyzed using the Chi-square test or Fisher exact test, while continuous variables were analyzed using analysis of variance (ANOVA), repeated measure analysis, and t-test. Within-group comparisons were made using Tukey's post-hoc analysis. Statistical significance was set at p<0.05.

## RESULTS

**Figure 1:**
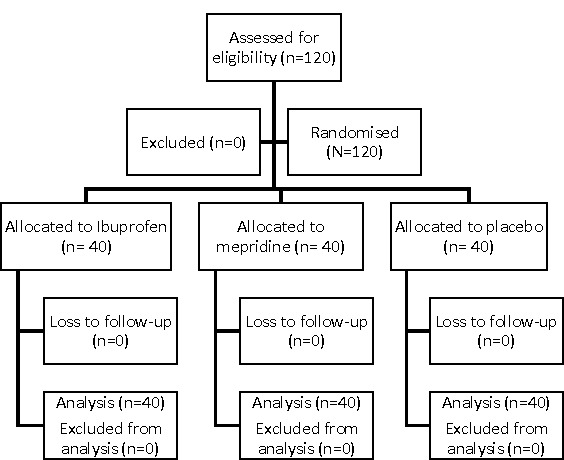
CONSORT flow diagram of patients in the study

A total of 120 eligible patients were enrolled in the study, and no patients were excluded. All enrolled patients completed the trial and were included in the analysis. The flow of patients throughout the study is presented in a Consolidated Standards of Reporting Trials (CONSORT) flow diagram (Figure 1).

According to demographic data for the 3 study groups, no differences were determined between groups in terms of sex, age, weight, surgery duration, and ASA physical status (p>0.05) (Table 3).

**Table 3:** Demographic characteristics of study participants (n=120)

Variable		groups			p-value
		Meperidine (n=40)	Ibuprofen (IV) (n=40)	Placebo (n=40)	
**Sex**	**Male**	10(25%)	8(20%)	6(15%)	0.53
	**Female**	30(75%)	32(80%)	34(85%)	
**ASA**	**ASA1**	34(85%)	35 (78.5%)	37(92.5%)	0.56
	**ASA2**	6(15%)	5(12.5%)	3(7.5%)	
**Age (year)**		40.5±10.5	35.4±9.1	39.2 ±10.1	0.06
**Weight (Kg)**		76.2±11.2	72.8±12.5	69.8±12.4	0.06
**Surgery duration (min)**		45.8±10.4	43.3±10.5	50.3±16.6	0.052

The incidence of shivering after surgery was 28.2% in the ibuprofen group, 23.7% in the meperidine group, and 35.9% in the placebo group, which was not statistically significant (p=0.72).

**Figure 2:**
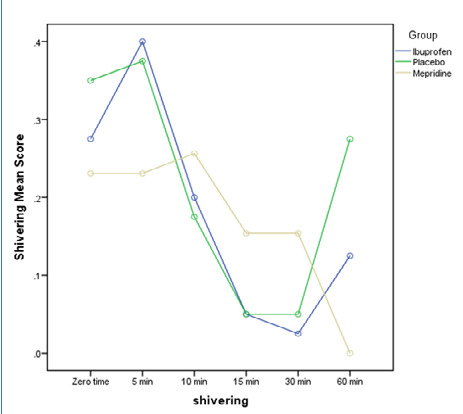
Mean shivering score after surgery in the recovery room

After entering the recovery room, no significant differences in shivering intensity were found between the groups (p=0.22). Similarly, the grade of shivering at all evaluation time points (5, 10, 15, 30, and 60 minutes after entering the recovery room) did not show any significant differences among the three groups (p=0.87) (Figure 2).

**Figure 3:**
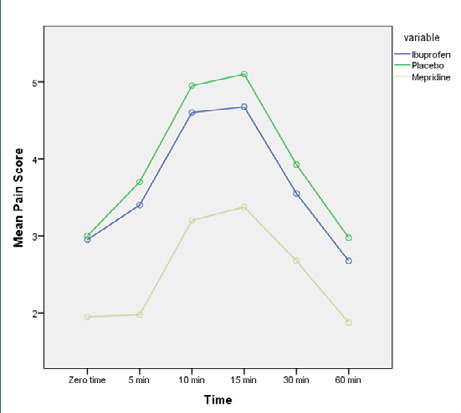
Mean pain score after surgery in the recovery room

VAS pain scores were assessed upon entering the recovery room. The results revealed a statistically significant difference between the groups, with the meperidine group exhibiting lower pain scores (ranging from 1.88±0.93 to 3.38±2.12) compared to the ibuprofen group (ranging from 2.68±0.83 to 4.68±2.1) and placebo group (ranging from 2.98±1.6 to 5.1±2.1). In all three groups, postoperative pain intensity increased up to 15 minutes after entering the recovery room, followed by a subsequent decrease (Figure 3).

**Figure 4:**
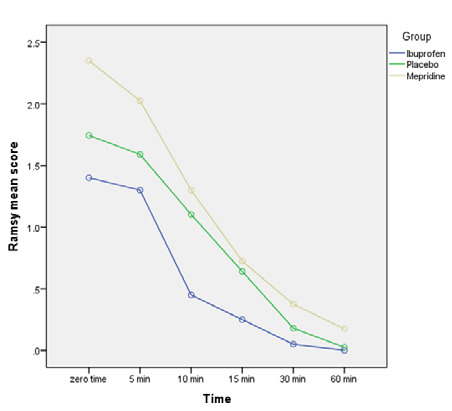
Sedation score in groups at postoperative time points

At all time points, the Ramsay sedation score was consistently highest in the meperidine group and lowest in the ibuprofen group. The difference in sedation scores between the groups was not statistically significant except at the 60-minute time point (p=0.066) after recovery (Figure 4). Over the one-hour study period, 40% of patients (n=16) in the meperidine group, no patients in the placebo group, and 10% of patients (n=4) in the ibuprofen group had a sedation score ≥4. This difference was found to be significant between the ibuprofen and meperidine group (p=0.02) and also between the meperidine and placebo group (p=0.01).

The incidence rate of nausea was 7.5% in the ibuprofen group, 20% in the meperidine group, and 0.0% in the placebo group. This difference was significant only at the 15-minute mark after entering the recovery room (p=0.025). Four patients in the ibuprofen group (10%), 6 patients in the placebo group (15%), and 5 patients in the meperidine group (12.5%) experienced vomiting. However, there was no significant difference in the incidence of vomiting among these groups (p=0.75).

## DISCUSSION

Reducing the side effects of opiates, such as respiratory dysfunction, nausea and vomiting, and sedation, is very important in patients after surgery. Shivering, a common and harmful complication of general anesthesia, can lead to hypoxia, pain, and lactic acidosis and interfere with monitoring ECG, blood pressure, and oxygen saturation [20, 21]. In this clinical trial, we compared the incidence of shivering, analgesic effect, sedation effect, and side effects of intravenous ibuprofen and meperidine in patients undergoing laparoscopic cholecystectomy.

Our study findings show a comparable incidence of shivering when using intravenous ibuprofen or meperidine. We searched for similar studies but found no investigations specifically examining intravenous ibuprofen as an anti-shivering drug. Therefore, we compared the results of studies investigating intravenous drugs with a similar mechanism of action as ibuprofen, such as cyclooxygenase inhibition.

Consistent with our study, Khezri *et al*. showed no difference between meperidine and ketorolac in preventing postoperative shivering [3]. This result is also consistent with another study indicating the potential effect of diclofenac sodium suppositories in decreasing postoperative shivering [22].

The exact anti-shivering effect of nonsteroidal anti-inflammatory drugs (NSAIDs) is still unknown. Prostaglandin E2, one of the main mediators of the hypothalamus, causes shivering after anesthesia via a central thermoregulatory pathway. This mediator is stimulated by COX-2 in the brain [23]. COX-2 inhibitors, such as ibuprofen, may affect perioperative thermoregulation by inhibiting COX-2, thus allowing the body to avoid a shivering response. The inhibitory effects of ibuprofen on pro-inflammatory mechanisms result in dampened release of pro-inflammatory cytokines during surgery, an excess of which might lead to shivering throughout surgery [23].

Despite the low intensity of pain in laparoscopic surgery compared to open surgery, treating pain after laparoscopic surgery is a clinical and research dilemma and a challenge. Postoperative pain can lead to prolonged hospital stays and delayed recovery after laparoscopic cholecystectomy [24]. In our study, the range of VAS scores in the placebo group during the study period (2.98±1.6 to 5.1±2.1) was consistent with previous studies on laparoscopic cholecystectomy [25]. In previous studies, intravenous ibuprofen has been approved for managing postoperative and non-surgical pain [26, 27]. The analgesic effect of ibuprofen is induced by COX-2 inhibition [28]. In our study, the pain intensity in the meperidine group was significantly lower than in other groups throughout the entire study period. However, when compared to the placebo, intravenous ibuprofen at a dose of 800 mg resulted in a nonsignificant decrease in pain intensity throughout the study period.

In contrast to the results of our study, previous studies have demonstrated statistically significant differences [29]. For example, Ahiskalioglu *et al*. [16] conducted a study in which preemptive administration of 400mg IV ibuprofen was followed by postoperative analgesia using IV paracetamol. This discrepancy may be attributed to differences in the method of intervention administration and dose variability among studies.

In our study, the ibuprofen group showed comparable or better control of nausea and vomiting than other groups, while the meperidine group had a higher incidence of nausea (20%) and vomiting (12.5%). This finding is consistent with published data in other studies. For example, the study by Southworth *et al*. [30] compared intravenous ibuprofen 400 mg and 800 mg following orthopedic or abdominal surgery to a placebo group. Nausea and vomiting in the intravenous ibuprofen group were significantly lower than in the control group. Similarly, Rosenblum *et al*. demonstrated that ibuprofen resulted in less nausea and vomiting than fentanyl in controlling pain after laparoscopic surgery [31]. Opioids, such as meperidine, can cause relaxation of the lower esophageal sphincter and delay gastric emptying through various mechanisms [32].

According to our findings, patients in the intravenous ibuprofen group experienced significantly less sedation compared to those who received meperidine and placebo. This difference was statistically significant at all time points of the study, except at the 60-minute mark.

A meta-analysis of 12 randomized controlled trials involving 994 patients who received parecoxib for postoperative pain management compared to those who received placebo or standard treatment analgesia, found lower incidence rates of nausea, vomiting, and early (1-hour) postoperative sedation scores in the parecoxib group [33]. Our study found no significant increase in side effects with a single dose of intravenous ibuprofen.

Some limitations of our study include its short duration, which may limit the generalization of the results. Further studies with longer follow-ups are recommended to confirm our findings. Additionally, a single dose of 800 mg of ibuprofen, regardless of patient weight, and its administration only 30 minutes before the end of surgery (according to our study design) may be considered a potential criticism. Another limitation is the lack of assessment of anesthesia duration, hospital stay, and cost-effectiveness. Moreover, the results may not be generalizable to other types of surgeries. Further studies are needed to explore the optimal dosing regimen of intravenous ibuprofen to prevent postoperative shivering and its potential therapeutic effect on postoperative shivering.

## CONCLUSION

In conclusion, according to the results of the present study, 800 mg intravenous ibuprofen appears to be an effective and low-risk intervention for the prevention of postoperative shivering after general anesthesia of laparoscopic cholecystectomy (by reducing the side effects associated with opioids such as sedation, nausea, and vomiting). However, 30 mg of intravenous meperidine was found to be a more effective analgesic compared to intravenous ibuprofen. Nonetheless, for patients who encounter challenges with meperidine administration, intravenous ibuprofen can serve as a suitable alternative for controlling postoperative shivering.

## Personal thanks

The authors are very thankful to the Research Deputy of Ardabil University of Medical Sciences due to their financial support regarding this study
